# Effective Enhancement of Water Absorbency of Itaconic Acid Based-Superabsorbent Polymer via Tunable Surface—Crosslinking

**DOI:** 10.3390/polym13162782

**Published:** 2021-08-19

**Authors:** Yong-Rok Kwon, Jung-Soo Kim, Dong-Hyun Kim

**Affiliations:** 1 Material & Component Convergence R&D Department, Korea Institute of Industrial Technology (KITECH), Ansansi 15588, Korea; yongrok@kitech.re.kr (Y.-R.K.); kimjungsoo11@kitech.re.kr (J.-S.K.); 2 Department of Material Chemical Engineering, Hanyang University, Ansansi 15588, Korea

**Keywords:** superabsorbent polymer, surface crosslinking, absorbency under load, centrifuge retention capacity, itaconic acid

## Abstract

A superabsorbent polymer (SAP) was synthesized by copolymerizing itaconic acid and vinyl sulfonic acid. The typically low absorbency of itaconic acid-based SAPs under mechanical loads was improved by introducing surface crosslinking. Fourier-transform infrared spectroscopy and X-ray photoelectron spectroscopy were used to characterize the synthesis and surface-crosslinking reactions in the SAP. Various conditions for surface-crosslinking reactions, such as the surface-crosslinking solution, content of surface-crosslinking agent, and reaction temperature, were explored and correlated with the gel strength and absorption characteristics of the resulting SAP particles. The distilled water content in the surface-crosslinking solution strongly influenced the absorption capacity of the SAP, but this sensitivity decreased when acetone was used as a co-solvent. Itaconic acid-based SAP that was crosslinked under optimal conditions exhibited centrifuge retention capacity and absorbency under a load of 31.1 and 20.2, respectively.

## 1. Introduction

Superabsorbent polymers (SAP) are a type of hydrogel formed by weak physical or chemical crosslinking [1]. Crosslinking molecules in the SAP prevent the material from dissolving in water or aqueous solutions and allow it to swell [2]. SAPs can absorb and retain water or aqueous solutions at capacities of up to hundreds to thousands of times the dry weight of the SAP [3]. These properties make SAPs a widely used material in a variety of specialized applications, including agriculture, horticulture, wastewater treatment, drug delivery systems, and disposable personal hygiene products [4,5,6].

Most commercial SAPs are produced by using acrylic acid (AA) as the main ingredient. However, AA-based SAP can cause skin irritation (such as itching and dryness) upon contact, decompose slowly, and may contaminate soil [7].

Itaconic acid (IA) was proposed as an alternative monomer to AA to prepare biocompatible and environmentally friendly SAPs. IA is a dicarboxylic acid and is produced industrially by fermenting sugarcane, potatoes, and other carbohydrates with microorganisms [8,9]. However, the low polymerization activities of IA make it difficult to prepare SAPs by using IA alone. Hence, several studies reported SAP syntheses that use IA as a comonomer and focused on generating biodegradable IA-based SAP. In our previous study, an SAP was prepared by using vinyl sulfonic acid (VSA) as a comonomer in order to enhance the polymerization efficiency of IA and boost the absorption properties of the resulting SAP [10]. VSA has a high salt resistance, due to both its high ionization tendency and low salt sensitivity that ultimately yield SAPs with high absorption capacities for saline [11].

Optimized SAPs for use in diapers have balanced centrifuge retention capacity (CRC) and absorbency under load (AUL) to provide a reliable absorption performance [12]. Commercial SAPs commonly used in diapers have CRC values of approximately 30 g/g and an AUL of approximately 20 g/g. However, most biomass-based SAPs have limited uses due to their low gel strength. There are methods to increase the degree of crosslinking of the SAP network to improve AUL, but they often cause significant reductions in CRC, which degrades the water retention capacity [13]. Thus, in order to address the trade-off between CRC and AUL, researchers have applied surface crosslinking (core-shell type) to increase the external surface-crosslinking density while maintaining a relatively lower crosslinking density within the volume of the SAP, thus retaining its water retention capacity [14,15,16,17]. According to their research, the content of the surface-crosslinking agent, reaction temperature, and thickness of the crosslinked layer are important conditions for controlling surface crosslinking [18,19]. In a previous study, we compared the absorption properties of IA-based SAPs that were surface-crosslinked by different crosslinking agents including diol, triol, diglycidyl ether, and ethylene carbonate (EC). This study established that EC was the most effective for enhancing AUL in the resulting SAPs [20].

To the best of our knowledge, there are few reported studies that investigate IA-based SAPs, especially those that provide a detailed understanding of surface-crosslinking factors. In this study, various factors that influence the surface crosslinking of SAPs, such as the solvent ratio of the surface-crosslinking solution, content of surface-crosslinking agent, and reaction temperature, were examined and correlated with the SAP absorbent performance. These interrogations ultimately suggested the best conditions to prepare SAPs for use in diapers. An SAP was prepared by using IA and VSA as comonomers and tetra (ethylene glycol) diacrylate as a crosslinking agent. Before and after introducing the surface crosslinking, the SAPs were characterized by Fourier-transform infrared spectroscopy (FTIR), X-ray photoelectron spectroscopy (XPS), thermogravimetric analysis (TGA), differential scanning calorimetry (DSC), and scanning electron microscope (SEM). The absorption properties of the resulting SAPs were evaluated in order to establish values for the CRC and AUL. In addition, the improved SAP gel strength was confirmed through rheological analysis.

## 2. Materials and Methods

### 2.1. Materials

IA (Junsei Chemical, Tokyo, Japan) and VSA (Sigma Aldrich, St. Louis, MI, USA) were used as monomers, while sodium hydroxide (NaOH, Samchun Pure Chemical, Pyeongtaek, Korea) was used as a neutralizing agent. Ammonium persulfate (APS, Sigma Aldrich, St. Louis, MI, USA) served as the initiator. Tetra (ethylene glycol) diacrylate (TTEGDA, Sigma Aldrich, St. Louis, MI, USA) and ethylene carbonate (EC, Sigma Aldrich, St. Louis, MI, USA) were used as the internal and surface-crosslinking agents, respectively. Methanol, ethanol, and acetone (Sigma Aldrich, St. Louis, MI, USA) were analytical grade and used as received.

### 2.2. Synthesis of Core-SAP (CSAP)

CSAP was synthesized by radical polymerization in aqueous solution. Table 1 reports the amount of each ingredient required for the CSAP. IA and VSA were added to a 500 mL 4-neck flask at a molar ratio of 7:3, after which 40 mL distilled water (DW) was added. NaOH was added for 1 h under a N_2_ atmosphere to neutralize 40% of the monomer. Subsequently, TTEGDA (0.5 wt%) was then added and the mixture was stirred for 30 min while maintaining the internal temperature of the flask at 60 °C. APS (1 wt%) was dissolved in 10 mL DW and then added to the mixture. Radical polymerization proceeded for 2 h after the initiator was added, and the temperature of the hot plate was adjusted to maintain the polymerization temperature at 60 °C. The resulting gel polymers were then annealed at 10 °C in a convection oven for 24 h and subsequently dried at 60 °C in a vacuum oven until there were no further changes in recorded weight. The dried samples were crushed in a mixer and classified into particle sizes of 300–600 μm.

### 2.3. Preparation of Surface-Crosslinked CSAP (SSAP)

The surface-crosslinking solution was prepared by dissolving EC in a mixture of various quantities of DW and acetone (10 mL). Surface crosslinking of CSAP was performed by infiltrating the prepared surface-crosslinking solution into the CSAP. The mixture was stirred and then transferred to an aluminum tray, where it was subsequently thinly spread, reacted at 100–160 °C, and washed with acetone. The resulting surface-crosslinked CSAP is designated SSAP. Sample names and surface-crosslinking conditions (the volume ratio of acetone and DW, the content of surface-crosslinking agent, reaction temperature, and reaction time) are shown in Table 2. The SSAP samples were named considering three variables. In the nomenclature, A, EC, and T mean acetone, surface-crosslinking agent, and temperature, respectively. In addition, the numbers after them indicate the volume ratio of acetone in the surface-crosslinking solution, weight percentage of the surface-crosslinking agent, and degree Celsius, respectively.

### 2.4. Characterization

The structures of CSAP and SSAP_A85 were analyzed by FTIR (Nicolet, Thermo Fisher Scientific, Waltham, MA, USA) using potassium bromide (KBr) pellets and a scan range of 400–4000 cm^−1^. The compositions of the SAPs were investigated by XPS (Nexsa, Thermo Fisher Scientific, Waltham, MA, USA) with a microfocus monochromatic X-ray source (Al-Kα at 1486.6 eV), a hemispherical analyzer, and a multichannel detector. TGA was carried out using a thermogravimetric analyzer (TA-2000, Dupont Co., Wilmington, NC, USA) under N_2_ atmosphere with a heating rate of 20 °C/min over 30–800 °C. The thermal behaviors of SAPs were investigated on a DSC (Q100, TA Instruments, New Castle, DE, USA). Two cycles of heating and cooling runs were adopted in the temperature ranges 30–150 °C and 30–230 °C, under N_2_ atmosphere at a heating rate of 10 °C/min. Glass transition temperature (T_g_) was determined at the midpoint of the glass transition after the second scan. The surface morphologies of SSAPs were examined using SEM (SU8010, Hitachi, Tokyo, Japan). Gold-coated SSAPs were analyzed at an acceleration voltage of 10 kV.

### 2.5. Gel Fraction

An SAP sample (0.5 g) was immersed in 500 mL DW and stirred for 48 h to extract unreacted monomers and oligomers. After that, the SAP was screened and dried in an oven at 60 °C until there was no change in weight. The gel content is calculated as follows:

(1) $$\mathrm{Gel}\;\mathrm{fraction}=\frac{\omega_{e}}{\omega_{i}}\times 100,$$

where ω_i_ and ω_e_ are the weight of initial and extracted dried SAP, respectively.

### 2.6. Rheological Analysis

The rheological measurements were performed using a rotational rheometer (TA instrument Ltd., ARES-G2) with parallel 25 mm diameter plates separated by a 3 mm gap. An SAP sample (0.2 g) was added to DW (10 mL) and allowed to swell for sufficient time (30 min) to absorb all of the DW. The expanded gel particles were then placed on the parallel plate and the rheological properties were measured at 25 °C. The storage modulus (G′) and loss modulus (G″) were recorded at constant shear strain of 0.2% in the frequency range of 0.1−100 Hz.

### 2.7. Free Absorbency (FA)

An SAP sample (0.1 g) was placed in a 100 mesh tea bag and allowed to swell in excess solvent (methanol, ethanol, or acetone) at room temperature until swelling equilibrium was reached. Then, the tea bag was removed from the solvent, excess solvent was wiped off, and the bag was weighed. The FA was calculated by using Equation (1) below, using data acquired under the aforementioned conditions to enable comparisons of the absorption properties of the SAPs under various conditions,

(2) $$\mathrm{FA}=\frac{\omega_{1}-\omega_{0}}{\omega_{0}},$$

where ω_1_ and ω_0_ are the weights of the swollen and dry SAP, respectively [21].

### 2.8. Swelling Kinetics

Swelling kinetics were evaluated to establish absorption equilibrium, absorption rate, and swelling capacity of the CSAP. Dried SAP sample (0.2 g) was added to the pre-wetted tea bag and immersed in various ratios of surface-crosslinking solutions. The mass was measured after absorbing excess water from the surface of the tea bag by using a paper towel at 5 min intervals. For all samples, the swelling ratio was calculated by using Equation (3):

(3) $$\mathrm{Swelling}\;\mathrm{ratio}=\frac{\omega_{1}-\omega_{0}}{\omega_{0}},$$

where ω_1_ and ω_0_ are the weights of the swollen and dry SAPs, respectively.

### 2.9. Centrifuge Retention Capacity (CRC)

CRC is a value that indicates the amount of water remaining after dewatering a swollen SAP via centrifugation. An SAP sample (0.1 g) was left to swell for 30 min in an aqueous solution containing 0.9 wt% of sodium chloride (NaCl) and then centrifuged at 300× *g*. The CRC was then calculated by using Equation (4):

(4) $$\mathrm{CRC}=\frac{\omega_{1}-\omega_{0}}{\omega_{0}},$$

where ω_1_ and ω_0_ are the weights of the swollen and dry SAP, respectively [22].

### 2.10. Absorbency under Load (AUL)

AUL is a value that represents the amount of water that an SAP can absorb under a certain pressure. A total of 0.16 g of SAP was uniformly distributed in a cylinder and then weighed and measured under a weight that supported a pressure of 0.3 psi. A ceramic filter plate was placed on a chalet and a filter paper was laid out. The prepared cylinder was placed on the filter paper, and a 0.9 wt% NaCl aqueous solution was added until the ceramic filter plate was sufficiently wetted. After 60 min, the cylinder was removed and weighed. The AUL was calculated by using Equation (5):

(5) $$\mathrm{AUL}=\frac{\omega_{1}-\omega_{0}}{\omega_{0}},$$

where ω_1_ and ω_0_ are the weights of the swollen and dry SAPs, respectively [23].

## 3. Results and Discussion

### 3.1. Preparation of CSAPs and SSAPs

**Scheme 1:**
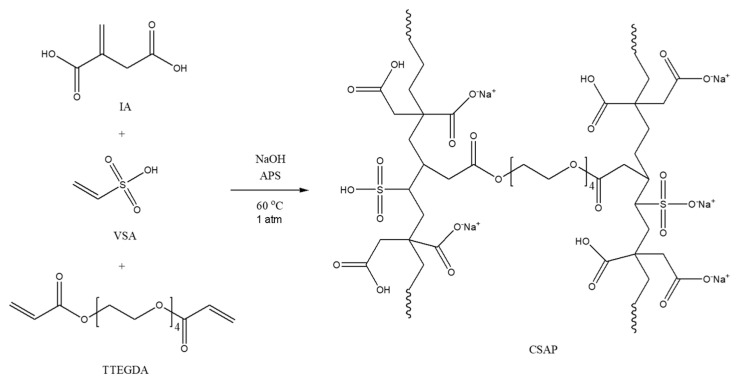
Synthetic procedure of CSAP and corresponding molecular structures of reagents and products.

The CSAP materials were prepared by crosslinking IA and VSA copolymers with TTEGDA. Scheme 1 shows the proposed structure of CSAP based on the chemical crosslinking reactions among IA, VSA, and TTEGDA. Prior to polymerization, the degrees of neutralization of the carboxyl group of IA and the sulfonyl group of VSA were 40%, which were obtained by the use of NaOH. Radicals produced by APS dissociation in water were added to one side of the double bond and formed an unpaired spin on the opposite side of the vinyl bond. The other double bonds were continuously attacked, resulting in polymerization. The double bond on both sides of TTEGDA caused the resulting CSAP to have a water-insoluble network structure. The gel fraction of the prepared CSAP was 91%. In general, IA has low activity in free radical polymerization, making it difficult to obtain IA homopolymers with a high conversion and molecular weight. However, since IA is more reactive in copolymerization with other monomers, CSAP with VSA as a comonomer showed a high gel fraction [24].

**Scheme 2:**
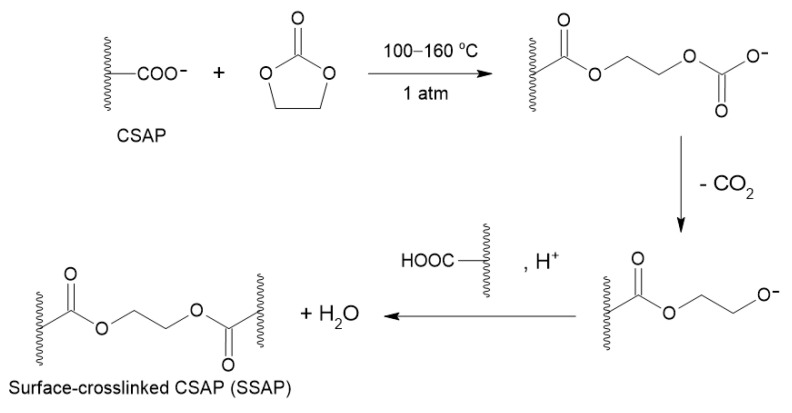
Surface-crosslinking reaction of CSAP by EC.

Scheme 2 shows the process for preparing SSAP by the chemical reaction of CSAP and EC. EC was dissolved in a mixture of acetone and DW prior to penetration into the CSAP. The CSAP absorbs DW but not acetone, which are polar and non-polar solvents, respectively. Based on polarity, the surface-crosslinking solution penetrated into the CSAP surface only to a shallow depth. The EC crosslinks the surface of CSAP by reacting with the two carboxyl moieties of the CSAP to form ester groups. The alkylene carbon of the EC undergoes nucleophilic attack by the ionized carboxyl group and subsequently reacts with another carboxyl group after carbon dioxide is released.

### 3.2. Characterization of CSAP and SSAP_A85

#### 3.2.1. FTIR Analysis

**Figure 1:**
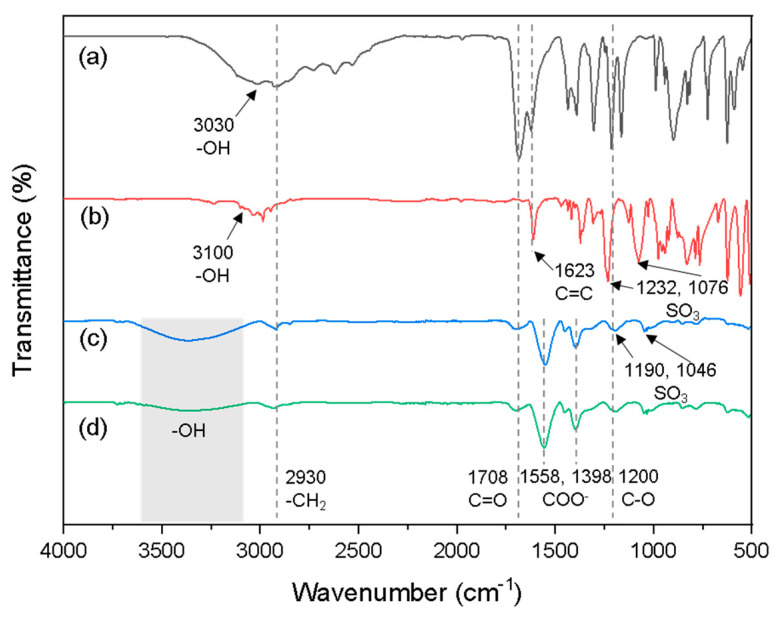
FTIR spectra of (**a**) IA, (**b**) VSA, (**c**) CSAP, and (**d**) SSAP_A85 materials.

The chemical structures of IA, VSA, CSAP, and SSAP_A85 materials were established by FTIR analysis (Figure 1). The peaks assigned to O-H vibrations of the carboxyl groups of IA at 3030 cm^−1^ and sulfonyl groups of VSA at 3100 cm^−1^ shifted to 3200–3600 cm^−1^ in CSAP [25,26]. The reason is that the vibration of O-H has a blue move due to the steric effect induced by the formation of the network structure [27]. The peaks at 1708 and 1200 cm^−1^ of IA are attributed to C=O and C-O, respectively, and the same was observed in CSAP [28]. The peaks at 1220 and 1045 cm^−1^ in the VSA spectrum arise from asymmetric and symmetric stretching oscillations of the SO_3_ group, and they were identified at 1190 and 1046 cm^−1^ in CSAP [29]. The FTIR spectrum of CSAP shows new characteristic absorption bands at 1558 and 1398 cm^−1^, attributed to C=O asymmetric stretching and symmetric stretching mode of carboxylate anion (COO^-^), respectively [30]. Moreover, the peak assigned to the vibration of C=C of IA and VSA at 1623 cm^−1^ is very clear; however, it disappears in the spectrum of CSAP.

It is difficult to establish the structural differences between SSAP_A85 and CSAP because of the high structural similarities of the EC in the CSAP and the polymer network, as well as the fact that EC is used in small quantities. Thus, the intensity of the broad absorption band at 3200–3600 cm^−1^ that corresponds to carboxylic acid moieties was used to compare the CSAP and SSAP materials [10]. The CSAP material produced a relatively greater peak because the carboxyl groups were consumed by the reaction with EC. In conclusion, FTIR analyses establish the successful synthesis of CSAP materials and surface-crosslinking reactions in SSAP materials.

#### 3.2.2. XPS Analysis

**Figure 2:**
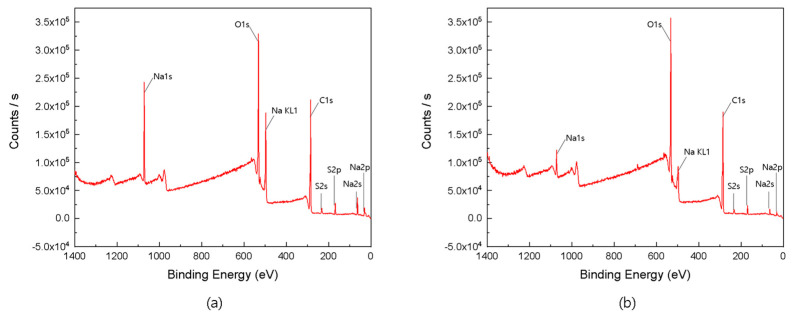
XPS spectra of (**a**) CSAP and (**b**) SSAP_A85 materials.

The XPS spectra of CSAP and SSAP_A85 materials are presented in Figure 2. The Na1s, Na2s, Na2p, O1s, Na KL1, and C1s peaks at 1072, 64, 31, 532, 498, and 286 eV, respectively, are observed for both samples. In addition, S2s and S2p peaks by the sulfonyl group of VSA are observed at 233 and 168 eV. The elemental compositions (at%) of the SAP surface obtained from the XPS analysis are listed in Table 3. Here, the atomic percentage of the element sodium is seen to decrease after surface crosslinking. The reason is that the carboxylate anion to which sodium ions can bind decreases due to the esterification reaction with EC. These results demonstrate the successful preparation of CSAP and SSAP_A85 materials.

#### 3.2.3. Thermal Properties

**Figure 3:**
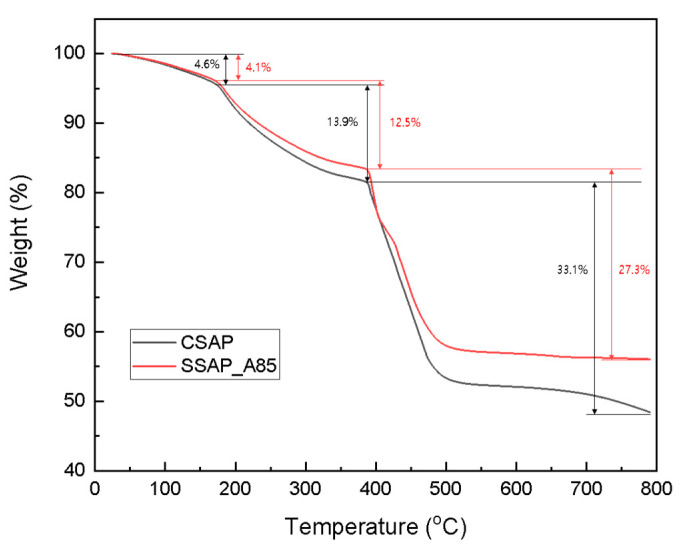
TGA curves of CSAP and SSAP_A85 materials.

The TGA curves of CSAP and SSAP_A85 materials are shown in Figure 3. CSAP and SSAP_A85 suffered weight loss in a similar temperature range. The weight loss of CSAP (4.6 wt%) and SSAP_A85 (4.1 wt%) at the initial stage below 169 °C is due to water loss. The reason for the weight loss at 169–386 °C was the removal of water molecules from two adjacent carboxyl groups in the polymer chain due to the formation of anhydrides. Here, the weight loss rate (12.5 wt%) of SSAP_A85 is lower than that of CSAP (13.9 wt%), which may be due to the consumption of carboxyl groups by the surface-crosslinking reaction. Above 386 °C, rapid weight loss is observed by the breakage of the polymer chain and the breakage of the crosslinked network structure. The total weight loss (43.9 wt%) of SSAP_A85 was lower than that of CSAP (51.6 wt%), indicating that surface crosslinking improves the thermal stability of SAP.

**Figure 4:**
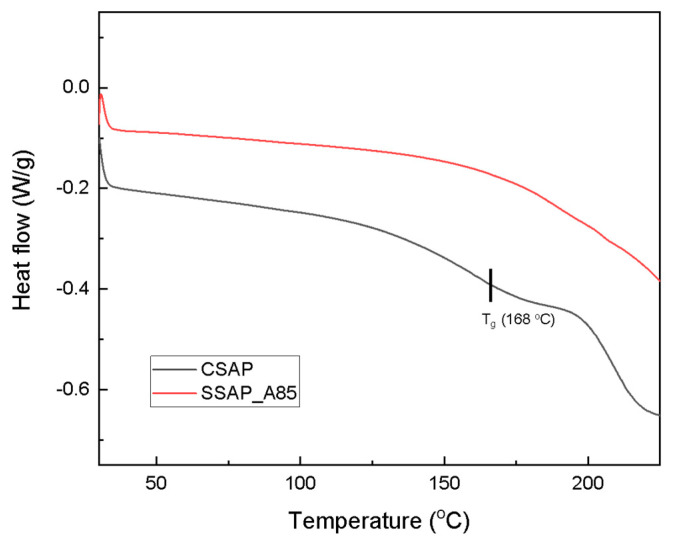
DSC thermograms of CSAP and SSAP_A85 materials.

Figure 4 shows the DSC thermograms of the CSAP and SSAP_A85 materials. CSAP showed a slope change corresponding to T_g_ at 168 °C, whereas the T_g_ of SSAP_A85 in the temperature range of 30–230 °C has not been determined. These results are attributed to an increase in crosslink density by surface crosslinking, which reduces the chain mobility of SAP, resulting in a less flexible network.

### 3.3. Swelling Behaviors of CSAP Materials in Mixtures of DW and Organic Solvent

**Figure 5:**
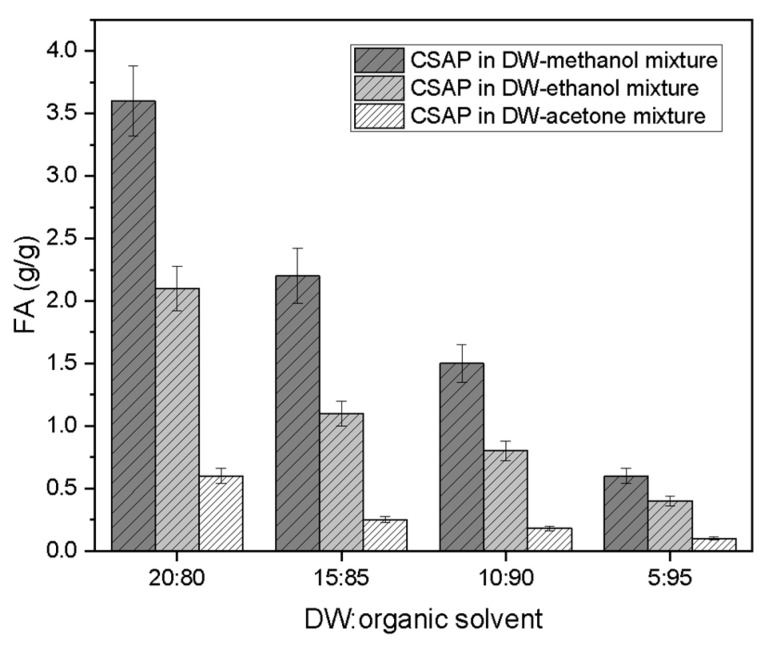
FA of CSAP exposed to solutions of DW and various organic solvents, including methanol, ethanol, or acetone, at various ratios.

The penetration depth of the surface-crosslinking agent should be limited to minimize reductions in the CRC and improve AUL. In general, SAPs have the greatest absorption in water. Water absorption of SAPs decreases with the addition of inorganic salts or organic solvents. In this way, in order to limit the penetration of the surface-crosslinking agent in the CSAP surface, the FA of CSAP was measured in various ratios of DW–methanol, ethanol, and acetone mixture. The results of these measurements are shown in Figure 5. Overall, the absorption capacity of CSAP decreased with increasing proportions of the organic solvent. The reduction in the overall absorption capacity was least to greatest for the following solvents in order: methanol, ethanol, and acetone. In particular, the DW–acetone mixture promoted extremely low FA values for CSAP vs. other mixtures. This can be interpreted based on differences in the solubility parameters of each organic solvent. The solubility parameter of water (δ_water_ = 23.2) is the largest among the solvents and is on the same order as methanol (δ_methanol_ = 14.5), ethanol (δ_ethanol_ = 12.7), and acetone (δ_acetone_ = 10.0) [26]. That is, the water absorption of CSAP decreases with the solubility parameter of the DW–organic-solvent mixture. The DW–acetone mixture was slightly absorbed by CSAP. Moreover, FA was the least sensitive to changes in the ratio of acetone and water. Thus, the use of the DW–acetone mixture as a solvent for the surface-crosslinking reaction yields a thin and uniform shell structure on the CSAP surface.

**Figure 6:**
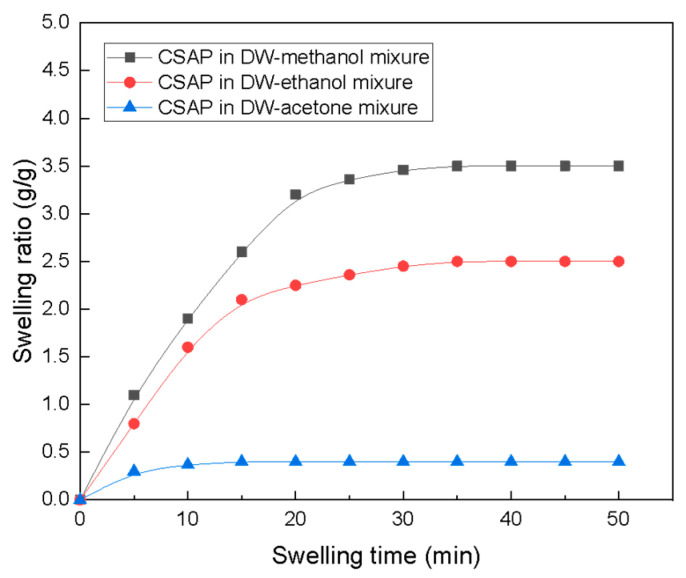
Swelling kinetics of CSAP in DW–organic-solvent solutions (volume ratio = 15:85).

The surface-crosslinking solution diffuses into the CSAP materials, which permits the swelling time to serve as a control of the thickness of the crosslinked layer. Importantly, this process depends on the time required for CSAP materials to reach an absorption equilibrium with the surface-crosslinking solution. Figure 6 shows the swelling kinetics of the CSAP materials exposed to various mixtures with a volume ratio of DW and organic solvent of 20:80. The DW–methanol and DW–ethanol mixture required more than 30 min to reach absorption equilibrium, whereas the DW–acetone mixture reached absorption equilibrium within 15 min. These results mean that the use of a mixture of methanol or ethanol can reduce the efficiency of the surface-crosslinking process of CSAP. In addition, long swelling times can cause an excessive diffusion of the surface-crosslinking agent into the CSAP core. These phenomena promote a blurred boundary between the core and shell of the SSAP. Thus, the DW–acetone mixture was selected as the solvent for the surface-crosslinking solution, and the swelling time of CSAP prior to surface crosslinking was fixed at 15 min.

### 3.4. Effect of the Volume Ratio of DW to Acetone on SSAPs

#### 3.4.1. SEM Analysis of CSAP and SSAPs

**Figure 7:**
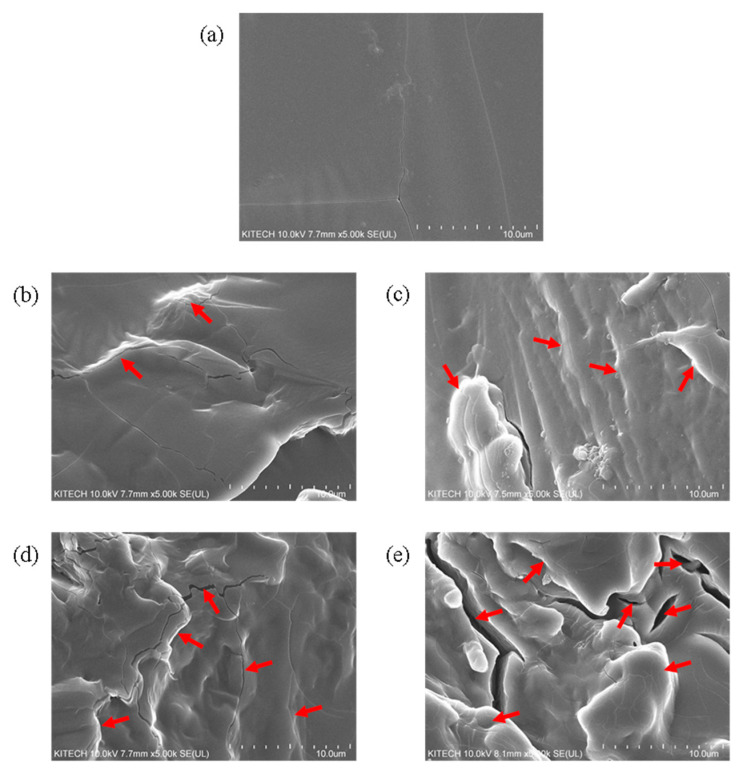
The SEM images of (**a**) CSAP, (**b**) SSAP_A95, (**c**) SSAP_A90, (**d**) SSAP_A85, and (**e**) SSAP_A80.

Figure 7 shows SEM images of CSAP and SSAP materials prepared with surface-crosslinking solutions of various DW to acetone volume ratios. A clean and smooth surface was observed on the CSAP. In the case of SSAP, as the acetone content in the surface-crosslinking solution decreased, the cracks and irregularities on the surface intensified. This change in surface properties is due to an increase in the surface-crosslinking density. The decrease in the acetone content allowed for the surface-crosslinking solution to penetrate more into the CSAP. After that, during the curing step, cracks and irregularities occurred on the surface because CSAP contracted through dehydration and crosslinking reactions.

#### 3.4.2. Rheological Properties of SSAPs

**Figure 8:**
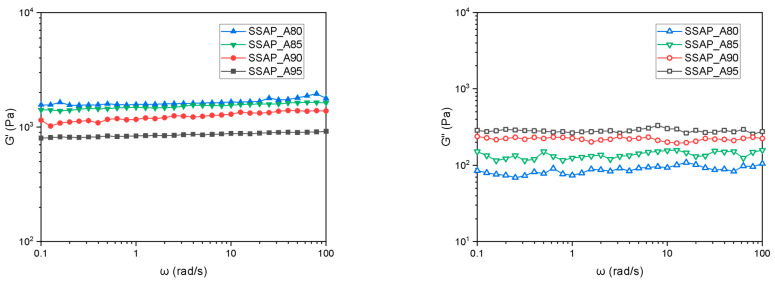
Storage modulus (G′) and loss modulus (G″) functions under frequency sweeps for SSAPs according to volume ratio of DW to acetone in the surface-crosslinking solution.

The gel strength of SSAPs prepared with surface-crosslinking solutions of various DW to acetone volume ratios is shown as a function of storage modulus (G′) vs. angle frequency in Figure 8. According to the Flory–Rehner correlation, the crosslinking density is directly related to G′ [27]. Therefore, since the decrease in acetone content increased the absorption of CSAP to the surface-crosslinking solution, the crosslinking density of SSAP increased. Consequently, the higher the polarity of the surface-crosslinking solution, the higher the gel strength and resistance to external stress of SSAP. On the other hand, the G″ of SSAPs showed the opposite trend. Generally, the increase in crosslink density leads to both a decrease in G″ by raising the elastic contribution and a reduction in the viscos contribution. This expectation is well met.

#### 3.4.3. Absorption Properties of SSAPs

**Figure 9:**
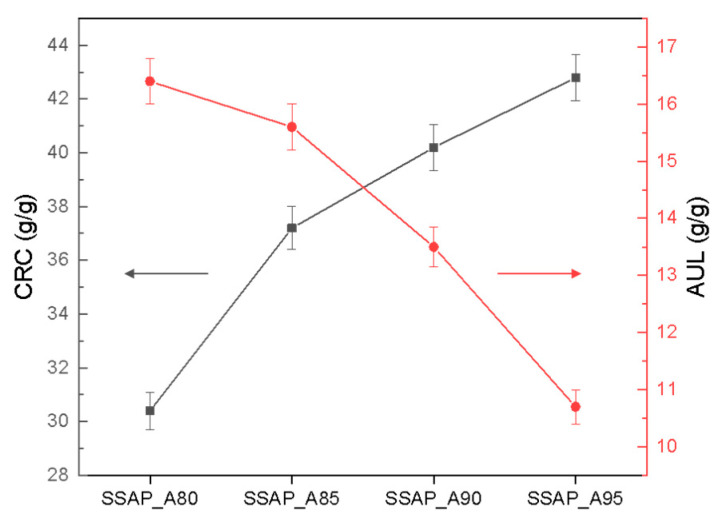
The CRC and AUL of SSAPs according to volume ratio of DW to acetone in the surface-crosslinking solution.

Figure 9 displays the effects of the DW to acetone volume ratio of the surface-crosslinking solution on the CRC and AUL of SSAP materials. All of the surface-crosslinking solutions had an EC content of 0.5 wt% (relative to the weight of the CSAP) whereas the acetone content was varied. Overall, increasing the acetone content concomitantly increased the CRC of the resulting SSAP, but decreased the AUL. This tendency arises because of the various amounts of the surface-crosslinking solution that were absorbed by CSAP. Solutions with lower ratios of acetone increase the solubility parameters of the surface-crosslinking solution, which promotes a larger quantity of the surface-crosslinking solution to be absorbed by the CSAP. Thus, the AUL of SSAP_A80, which has a low acetone ratio, had the largest quantity. Interestingly, when the acetone ratio was decreased from 85 to 80%, a sharp decline in the CRC was observed. In Figure 5, it is shown that the FA value decreased sharply at the 80–85% acetone ratio. This result means that the penetration of the surface-crosslinking solution varies considerably between these two ratios. The resulting thickness of the surface-crosslinking layer dramatically reduces the absorption capacity of the SSAP materials. The ratio of the surface-crosslinking solution used in the SSAP_A85 material was selected to prevent the rapid lowering of CRC.

### 3.5. Effects of Surface-Crosslinking Agent Content on Absorption Properties of SSAPs

**Figure 10:**
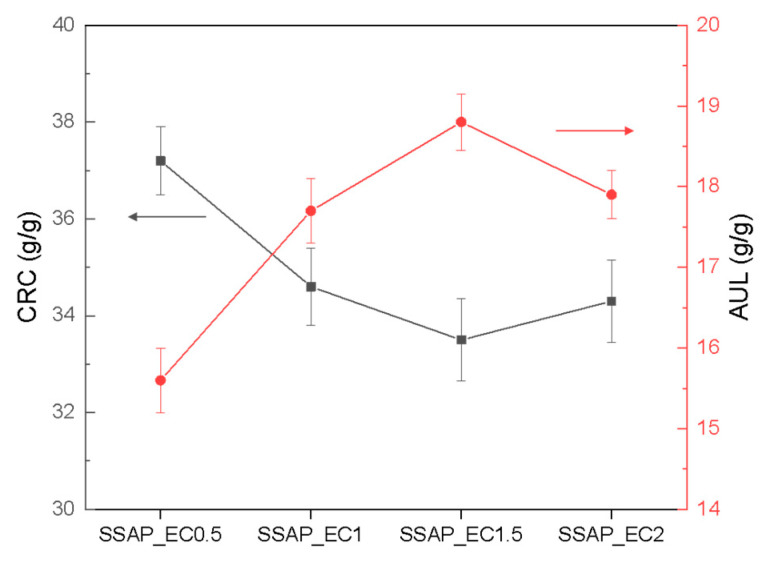
CRC and AUL of SSAP materials prepared with various contents of surface-crosslinking agent.

Figure 10 displays the relationships between the EC content and absorption properties of the resulting SSAPs. The crosslinking density primarily depends on the concentration of the surface-crosslinking agent. As the EC content increases from 0.5 to 1.5 wt%, the CRC decreases and AUL increases. AUL is an indirect indicator of gel strength, and an increase in the EC content improves the crosslinking density of the SAP surface and reduces the expansion rate of the overall polymer network. By comparison, as the EC content increases beyond 1.5 wt%, the CRC increases and the AUL decreases. This trend arises because excessive EC can cause side reactions. In Clements’ review, excess EC could undergo an alkylation reaction with the carboxylic acid vs. an esterification reaction [28]. In Scheme 2, the ideal surface-crosslinking reaction is the esterification of EC with two carboxylic acids. However, if an excessive amount of EC is added, incomplete surface crosslinking may occur due to alkylation reactions. As a result, an optimal EC concentration was established to be 1.5 wt%, and the resulting SSAP_EC1.5 material exhibited the best performances, with a CRC and AUL of 33.8 g/g and 18.8 g/g, respectively.

### 3.6. Effects of Temperature of Surface-Crosslinking Reaction on Absorption Properties of SSAPs

**Figure 11:**
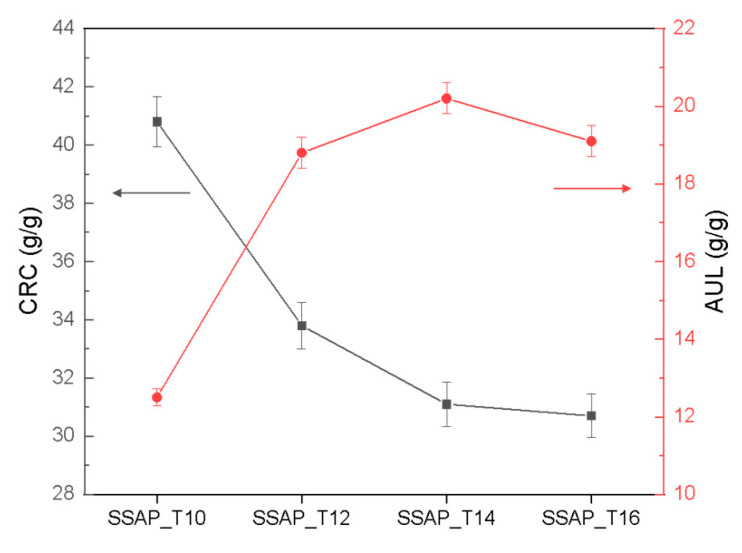
The CRC and AUL of SSAP materials prepared by using different temperatures for the surface-crosslinking reaction.

The temperature at which the surface-crosslinking reaction is conducted is one of the most important factors for the crosslinking reactions because it not only improves the reaction efficiency but also greatly affects the properties of SAP via deterioration. The absorption properties of the SSAP materials prepared by using various temperatures for the crosslinking reaction are shown in Figure 11. A sharp change in the CRC and AUL was observed between 100–120 °C. This result demonstrates that temperature conditions of 100 °C were insufficient to cure the surfaces of CSAP and SSAP_T10, which, as a result, had very low crosslinking densities. With increased reaction temperatures, the CRC of the SSAP materials gradually decreased. However, the AUL of the SSAP materials showed a different trend. The AUL of the SSAP materials increased with an increasing reaction temperature and then decreased again at 160 °C. Dehydration of the carboxylic acid groups is reported to occur at a temperature of 160 °C [29]. If such dehydration was intramolecular, the result would be the formation of cyclic anhydride; otherwise, the anhydride would act as a short crosslinking agent and reduce hydrophilicity. Moreover, this phenomenon could decrease the available carboxylic acid groups. Consequently, the surface-crosslinking efficiency for samples cured above 140 °C declines.

## 4. Conclusions

An IA-based eco-friendly SAP was synthesized by copolymerizing IA and VSA at a molar ratio of 7:3. The preparation of CSAP and SSAP polymeric materials was established by FTIR and XPS analyses. In this study, surface crosslinking was introduced in order to improve the relatively low AUL of IA-based SAPs, and optimal surface-crosslinking conditions were determined. Materials prepared with these optimal conditions had excellent absorption performances, with CRC values of 31.1 g/g and AUL values of 20.2 g/g.

Acetone, which has the lowest solubility parameter among hydrophilic organic solvents tested here, was selected as the optimal solvent because it limited the penetration of the surface-crosslinking agent into the CSAP materials. FA was the least sensitive to changes in the ratio of acetone and water, and so the DW–acetone mixture was suitable for forming a thin shell structure on the SSAP surfaces. As the volume ratio of acetone in the surface-crosslinking solution decreased, the gel strength of SSAP increased. When the surface-crosslinking agent, EC, was added at 1.5 wt%, based on the weight of the CSAP, the resulting SSAP materials exhibited the highest AUL values. Excessive EC content caused alkylation side reactions and reduced crosslinking efficiency. The reaction temperature of the surface crosslinking also greatly influenced the absorption performance of SSAP materials. As the reaction temperature increased, the CRC tended to decrease. The AUL was highest when the reaction temperature was 140 °C. In addition, SSAP had better thermal stability than CSAP.

This study established optimal surface-crosslinking conditions for an IA-based SAP, which specifically include the ratio of DW to acetone of 15:85 in the solvent, a surface-crosslinking agent amount of 1.5 wt%, and a reaction temperature of 140 °C. The relationships among these synthesis variables and corresponding absorption properties of the SAP materials are anticipated to broaden the application of IA-based SAPs.

## Figures and Tables

**Table 1:** Ingredients of the prepared CSAP.

Sample	IA (g)	VSA (g)	NaOH (g)	TTEGDA (g)	APS (g)
CSAP	72.86	49.74	21.76	0.73	1.47

**Table 2:** Conditions for surface-crosslinking reactions for CSAP.

Sample Name	CSAP (g)	DW: Acetone (Volume Ratio)	EC (wt%)	Temperature (°C)
SSAP_A80	3	20:80	0.5	120
SSAP_A85	3	15:85	0.5	120
SSAP_A90	3	10:90	0.5	120
SSAP_A95	3	5:95	0.5	120
SSAP_EC0.5	3	15:85	0.5	120
SSAP_EC1	3	15:85	1	120
SSAP_EC1.5	3	15:85	1.5	120
SSAP_EC2	3	15:85	2	120
SSAP_T100	3	15:85	1.5	100
SSAP_T120	3	15:85	1.5	120
SSAP_T140	3	15:85	1.5	140
SSAP_T160	3	15:85	1.5	160

**Table 3:** The elemental compositions of CSAP and SSAP_A85.

Sample Name	O1s (at%)	C1s (at%)	Na1s (at%)	Na KL1 (at%)	Na2s (at%)	Na2p (at%)	S2p (at%)
CSAP	28.39	49.10	7.06	5.41	3.92	4.21	1.89
SSAP_A85	33.91	58.64	1.77	1.35	1.00	1.05	2.26

## Data Availability

Data sharing not applicable.
